# The Future of Hospitals

**Published:** 1922-11

**Authors:** 


### THE FUTURE OF HOSPITALS.

IN the Annual Oration delivered before the Medical Society of London and published in the "Lancet," Mr. H. J. Waring, Surgeon to St. Bartholomew's and Dean of the Faculty of Medicine of the University of London, has made some important suggestions as to the future of hospitals. He points out that the recent discussions concerning the allocation of money contributed by patients and by public authorities have centred round the "rights" of the medical man, whilst the "rights" of the patients and of the hospitals have been left out. In our May issue we drew attention to this omission and endeavoured to remedy it by stating the case for the public and the hospitals. A great merit of Mr. Waring's paper is that, while as surgeon to a great hospital he knows thoroughly the medical side of the question, he has given full regard to rights and interests of patients.

In the past the benefits of the voluntary hospitals have been limited to the necessitous poor, whilst the doors have been closed to those who have provided, or contributed towards, the funds. Thus the necessitous poor have been much better provided for than the upper and middle classes, and public hospitals have been far better built and equipped than private hospitals. The public have during recent years become cognisant of this fact, and Mr. Waring has observed a gradually increasing demand for admission on the part of the non-necessitous. The laity have begun to recognise that the investigation, diagnosis, and treatment of disease with expedition, and the likelihood of obtaining the best result, can be satisfactorily carried out only in a properly built, equipped, and staffed institution, clinic, or hospital, and that, for the most part, so-called private hospitals and nursing homes are merely adapted second-class private dwellings. The scientific principles of cleanliness are better understood, and the internal conditions of hospitals known; people are beginning to realise that their prejudices against them are unfounded, and that they are likely to get better investigation and treatment there than elsewhere. This change of opinion has been expedited and assisted by the increasing number of people who live in tenement mansions, flats, or apartment houses.

At present satisfactory accommodation is provided in hospitals for those unable to pay anything and for those who can contribute towards their maintenance and cost of accommodation, but not for the middle classes who can pay for these and also moderate fees for treatment, or for the richer classes who can pay for better-class accommodation and ordinary fees for treatment. The buildings of most public hospitals are not suitable for provision of first-class accommodation of this kind by reconstruction. Moreover, in most instances the funds utilised have been either given or legated for distinctly charitable purposes. Our hospitals, as regards building arrangements and beds, are for the necessitous poor; they do not contain accommodation for the suffering rich. On the other hand, in many hospitals the special departments, such as those of pathology, electro-therapeutics, mechano-therapeutics, X-ray and actino-therapeutics have accommodation and equipment sufficient for all charitable purposes, and also for patients in a paying part of the hospital.

Mr. Waring considers that the equipment of a general hospital for the extra functions mentioned can be satisfactorily carried out only by building a new department or hospital block in the grounds of, or on land adjacent to and in direct connection with, the hospital. He has prepared plans of such an addition to a charitable hospital or public medical institution which would provide hospital requirements for all classes. He suggests that the "paying block" should be in close connection with the institution, because the staffs of the special departments are often willing, and have plenty of room and personnel, to carry on the investigations appertaining to a paying patients' department.

Mr. Waring points out the advantages which the various parties concerned would gain by the provision of such a department. Members of the upper and middle classes could readily obtain institutional investigation and treatment of the same high standard as that which they provide or assist in providing for the necessitous poor. The special departments of hospitals could be more fully and continuously utilised than at present, especially those open only on certain days in the week. The overhead charges for general administration would not necessarily be increased, and in any case would not be proportionately increased; a portion of such charges could legitimately be transferred to the paying department, which could thus be made a source of profit to the hospital. Members of hospital staffs could have some of their own patients in the private or paying department, and thus save considerable time in going from one private hospital or nursing home to another. Resident medical officers could be appointed to the paying department, whose services would be available in the absence of the patient's own medical men. The advantages of the department would be greatest in connection with maternity, and surgery. In this country there is a lamentable lack of satisfactory accommodation for maternity, and the mortality is little different from that of the pre-aseptic days—a serious reflection on our methods.

